# The Reduction of Tau Hyperphosphorylation by Cornel Iridoid Glycosides Is Mediated by Their Influence on Calpain Activity

**DOI:** 10.1155/2022/9213046

**Published:** 2022-01-20

**Authors:** Kaiwen Guo, Cuicui Yang, Lan Zhang

**Affiliations:** Department of Pharmacy, Xuanwu Hospital of Capital Medical University, National Clinical Research Center for Geriatric Diseases, Beijing Engineering Research Center for Nervous System Drugs, Beijing Institute for Brain Disorders, Key Laboratory for Neurodegenerative Diseases of Ministry of Education, 45 Chang-Chun Street, Beijing 100053, China

## Abstract

Alzheimer's disease (AD) is the most common type of dementia, and the abnormal hyperphosphorylation of the tau protein is the main component of its pathogenesis. Calpain was found to be abnormally activated in neurofibrillary tangles (NFTs) in a previous report. Cornel iridoid glycosides (CIG) have been reported to reduce the hyperphosphorylation of tau protein. Nevertheless, the role of calpain in the reduction tau hyperphosphorylation by CIG remains unclear. In the present study, we investigated the effect of CIG on calpain activity through in vitro and in vivo experiments. Western blotting results suggested that CIG decreased the phosphorylation of tau at Ser 404 and Ser 262 sites in P301S mice. Moreover, CIG inhibited the activity of calpain and glycogen synthase kinase 3*β* (GSK-3*β*) and enhanced the activity of protein phosphatase 2A (PP2A) both in vivo and in vitro. CIG also inhibited the activation of PP2A and reduced the GSK-3*β* activity caused by the calpain activator dibucaine. In addition, the main components of CIG, morroniside and loganin, play an equivalent role in reducing calpain activity, as the effect of their combined use is equivalent to that of CIG. The abovementioned findings revealed that CIG improved PP2A activity and reduced GSK-3*β* activity by adjusting the activity of calpain 1, leading to a reduction in the phosphorylation of tau. This study highlights the remarkable therapeutic potential of CIG for managing AD.

## 1. Introduction

The hyperphosphorylation of the tau protein causes microtubule breakdowns, leading to neuronal conduction system disorders and the formation of neurofibrillary tangles (NFTs), causing cognitive impairment [1, 2].

Reports analyzing stained brain tissue from Alzheimer's disease (AD) patients found that neurons containing NFTs showed high expression levels of the protein calpain [3]. The calpain family has 15 members; calpain 1 plays an important role in the activation of glycogen synthase kinase 3*β* (GSK-3*β*) and protein phosphatase 2A (PP2A) [4–6], and calpain 2 can also regulate the activity of PP2A [7]. Calpastatin, a calpain inhibitor, is the only endogenous specific calcium-activated neutral protease family inhibitor; calpain activity is strictly regulated by calpastatin in the presence of Ca^2+^ [8]. Alicapistat is a new drug being developed to treat AD by inhibiting calpain 1/2 activity, and trials were terminated due to inadequate CNS concentrations to obtain a pharmacodynamic effect [9]. Therefore, calpain may be a new target for AD treatment.

The phosphorylation of tau is regulated by phosphokinases and phosphatases [10]. PP2A is a serine/threonine protein phosphatase that dephosphorylates proteins in eukaryotes; the activity of PP2A in the brains of patients with AD is reduced significantly [11]. GSK-3 is a serine/threonine phosphokinase that is widely distributed in living organisms; GSK-3*β* is considered a key player in tau phosphorylation [12], and inhibiting its activity may revert learning and memory impairment [13]. The activities of GSK-3*β* and PP2A are influenced by calpain.

Cornel iridoid glycoside (CIG) was found in the literature to significantly inhibit abnormal hyperphosphorylation of tau protein, protect the structure of neuronal microtubules, reduce the formation of neurofibrillary tangles, and increase the number of surviving neurons in the cerebral cortex and hippocampus and was shown to remarkably improve the learning and memory function in AD animal models [14].

Based on these reports and our preliminary data, we examined whether CIG oral treatment can revert the hyperphosphorylation of tau protein and improve cognitive functions in AD mouse models by inhibiting calpain activity.

## 2. Materials and Methods

### 2.1. Animal Experiment

Ten-month-old P301S tau transgenic mice (B6; C3-Tg (Prnp-microtubule-associated protein tau (MAPT)^*∗*^P301S) Ps19Vle/JNju) were grown at the Nanjing Biomedical Research Institute of Nanjing University. The P301S mice were randomly divided into seven groups, with 15 mice in each group. Daily intragastric administration of CIG (50, 100, and 200 mg/kg) was performed for 3 months. They were housed in groups in standard individual ventilated cages (32 cm × 20 cm × 12.5 cm) containing wood shavings as litter at a controlled temperature of 20–22°C and a controlled humidity of 45%–55% on a modified 12/12 h dark-light cycle. Food and water were provided ad libitum. All efforts were made to minimize pain and suffering and to reduce the number of animals used. All experimental procedures were conducted in accordance with the Regulations of Chinese Experimental Animal Administration Legislation and were approved by the Bioethics Committee of Xuanwu Hospital of Capital Medical University.

### 2.2. Cell Culture

Neuro-2a (N2a) cells were obtained from the Institute of Materia Medica, CAMS, and PUMC and maintained in MEM supplemented with 10% fetal bovine serum (FBS, Gibco, Grand Island, NY, USA) and 1% penicillin-streptomycin solution (PS, Gibco) in a humidified incubator with 5% CO_2_ at 37°C.

MAPT transgenic cells (Biocytogen, Wakefield, MA, USA) were maintained in MEM supplemented with 10% fetal bovine serum (FBS, Gibco), 1% penicillin-streptomycin solution (PS, Gibco), and 0.06% puromycin (Beyotime, Jiangsu, China) in a humidified incubator with 5% CO_2_ at 37°C. To investigate the effects of CIG on neuronal pathology, N2a cells were cultured at 2.5 × 10^5^ cells/well in 6-well plates and treated with CIG (50, 100, and 200 *μ*g/mL) and its monomers morroniside (94 *μ*g/mL) and loganin (47 *μ*g/mL), for 24 h.

Another group of N2A cells were treated with the calpain activator dibucaine (200 *μ*M) for 6 h and then treated with CIG (50, 100, and 200 *μ*g/mL) and its monomers morroniside (94 *μ*g/mL) and loganin (47 *μ*g/mL), for 24 h.

### 2.3. Western Blotting

Protein samples were separated by SDS-PAGE and transferred to polyvinylidene fluoride membranes (Millipore, Burlington, MA, USA). Membranes were blocked in 5% skim milk for 1 h at 25°C and incubated with calpain 1 (1 : 1000, Abcam, Cambridge, MA, USA), calpain 2 (1 : 1000, Abcam), calpastatin (1 : 1000, Cell Signaling Technology, Danvers, MA, USA), and p-GSK3*β* (Ser 9) (1 : 1000, Cell Signaling Technology) overnight at 4°C. The membranes were then washed with TBST three times and incubated for 1 h with appropriate HRP-conjugated secondary antibodies at room temperature. Blots were washed three times with TBST, and chemiluminescence (ECL) was used to detect the bands when exposed to an X-ray film (Tanon, ABclonal, Woburn, Massachusetts, USA).

### 2.4. PP2A Activity

A serine threonine phosphatase assay system kit (Promega, Madison, WI, USA, V2460) was used to detect PP2A activity, following the manufacturer's instructions.

### 2.5. Statistical Analysis

All data are presented as mean ± standard error of mean (SEM). Multiple groups were tested using one-way analysis of variance (ANOVA) followed by the LSD test to determine the statistically significant groups, using SPSS 20 for data analysis. A *p* value <0.05 (^*∗*^) was considered to be statistically significant. GraphPad v.7.00 was used to draw the chart.

## 3. Results

### 3.1. Effect of CIG on the Phosphorylation of Tau in P301S Mice

**Figure 1:**
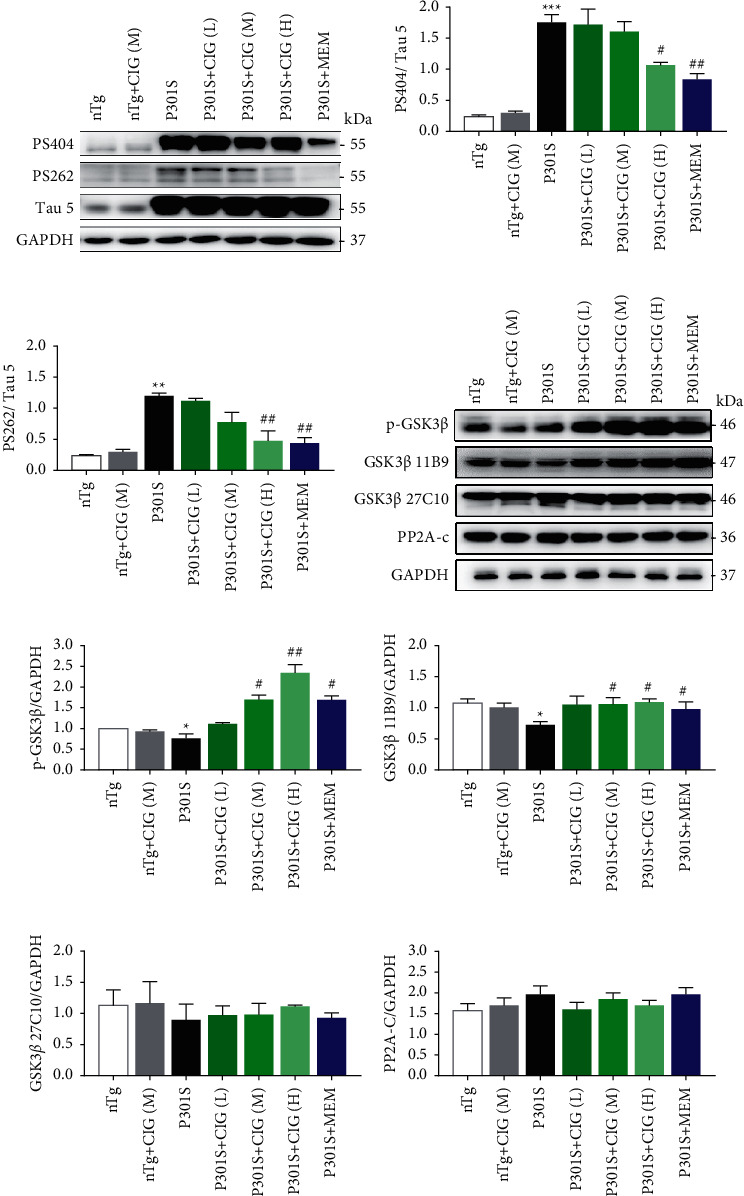
Effects of treatment with CIG on protein tau phosphorylation. (a) CIG reduced tau protein phosphorylation in P301S mice. P301S mice were treated with CIG and MEM everyday for 3 months. The expression of phosphorylation of tau at (b) Ser404, (c) Ser262, and GAPDH in P301S mice is shown. (d) The expression of phosphorylation of GSK-3*β* at (e) Ser 9, (f) N-terminal of GSK-3*β* (11B9), (g) GSK-3*β* 27C10, and (h) PP2A-c in P301S mice is shown. Mean ± SEM, *n* = 4; ^*∗*^*p* < 0.05, ^*∗∗*^*p* < 0.01, ^*∗∗∗*^*p* < 0.001, compared with the nTg group; #*p* < 0.05, ##*p* < 0.01, compared with the P301S group. CIG (L): 50 mg/kg, CIG (M): 100 mg/kg, and CIG (H): 200 mg/kg. MEM: memantine; CIG: cornel iridoid glycosides; GAPDH: glyceraldehyde 3-phosphate dehydrogenase; GSK-3*β*: glycogen synthase kinase 3*β*; and PP2A: protein phosphatase 2A.

We first examined the expression of tau in the P301S and nTg mice. We found a higher expression level of tau 5 in P301S mice than in nTg mice (Figure 1(a)). We further examined the level of tau phosphorylation in the seven groups: nTg, nTg treated with CIG (100 mg/kg), P301S mice, low-dose CIG (50 mg/kg)-treated P301S mice, middle-dose CIG (100 mg/kg)-treated P301S mice, high-dose CIG (200 mg/kg)-treated P301S mice, and MEM-treated P301S mice. As shown in Figure 1(a), CIG reduced the phosphorylation of tau protein at Ser 404 and Ser 262 sites. Furthermore, the reduction was dose dependent.

Figure 1(d) shows that CIG at different concentrations was found to reduce the previously high expression level of p-GSK-3*β*, with high-dose CIG being the most effective. However, CIG administration did not alter the truncation of GSK-3*β*. No significant difference in the expression level of PP2A-c was observed among the nTg mice, P301S mice, and CIG-treated P301S mice (Figure 1(h)).

### 3.2. CIG Inhibited Calpain Activity in P301S Mice

**Figure 2:**
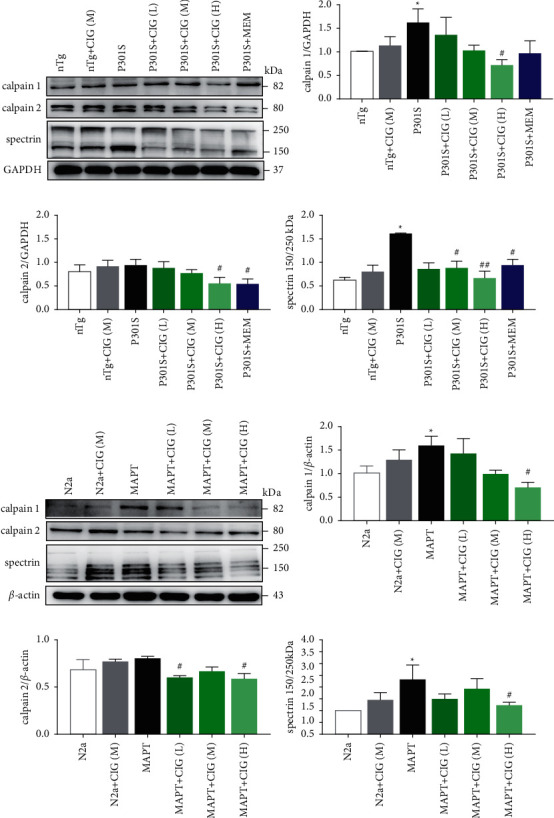
Effects of treatment with CIG on calpain activity. (a) CIG reduced calpain activity in P301S mice and MAPT cells. The expression of (b) calpain 1, (c) calpain 2, (d) spectrin, and GAPDH in P301S mice is shown. Mean ± SEM, *n* = 4; ^*∗*^*p* < 0.05, compared with the nTg group; #*p* < 0.05, ##*p* < 0.01, compared with the P301S group. MAPT cells were treated with CIG for 24 hours. (e) The expression of (f) calpain 1, (g) calpain 2, (h) spectrin, and GAPDH in MAPT cells is shown. Mean ± SEM, *n* = 3; ^*∗*^*p* < 0.05, compared with the N2a group; #*p* < 0.05, compared with the MAPT group. CIG (L): 50 *μ*g/mL, CIG (M): 100 *μ*g/mL, CIG (H): 200 *μ*g/mL, CIG: cornel iridoid glycosides, MAPT: microtubule-associated protein tau, and GAPDH: glyceraldehyde 3-phosphate dehydrogenase.

In initial studies, we analyzed the expression of calpain 1 and calpain 2 in the nTg, P301S, and P301S mice treated with CIG (50, 100, and 200 mg/kg). Western blot analyses demonstrated that the expression levels of calpain 1 and calpain 2 were downregulated after the high-dose CIG treatment compared to the levels in the salt-treated group (Figure 2(a)). The cleavage of spectrin *α* II from 250 kDa to 150 kDa, which is an important calpain activation marker, was significantly reduced after CIG administration (Figure 2(a)).

### 3.3. CIG Inhibited Calpain Activity in MAPT-Overexpressed N2a Cells

To verify whether CIG can reduce the activity of calpain, we used N2a cells with overexpressed MAPT for further experiments. N2a, MAPT, and CIG (50, 100, and 200 *μ*g/mL)-treated MAPT cells were used to detect the expression levels of calpain 1, calpain 2, and spectrin *α* II. As shown in Figure 2(e), calpain 1 and calpain 2 expression levels were significantly lower in high-dose CIG-treated cells than in MAPT cells. Cleavage of spectrin *α* II was decreased in the CIG-treated group compared to that in the model group (Figure 2(e)).

Taken together, these results indicate that CIG effectively attenuated calpain activity both in vivo and in vitro.

### 3.4. Morroniside and Loganin Inhibited Calpain Activity in MAPT-Overexpressed N2a Cells

**Figure 3:**
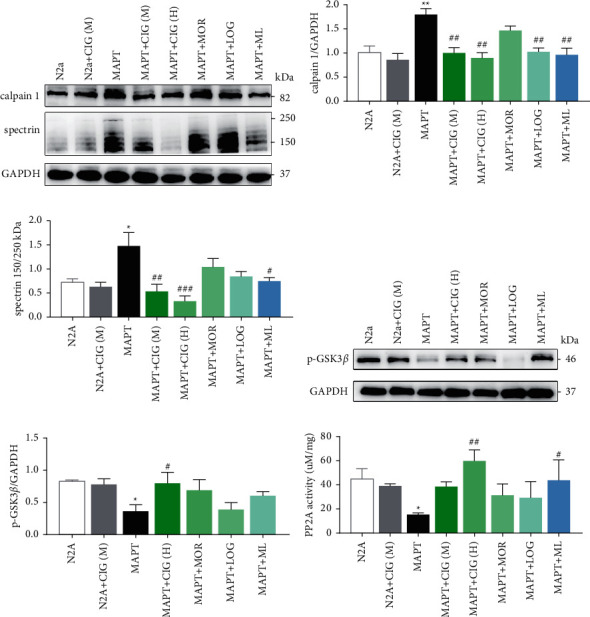
Effects of morroniside and loganin treatment on calpain activity. (a) CIG, morroniside, and loganin alleviated the activation of calpain in MAPT cells. The expression of (b) calpain 1, (c) spectrin *α* II, (e) phosphorylation of GSK-3*β* at Ser 9, and GAPDH in MAPT cells was determined by western blot and semiquantitative analysis. (f) The activity of PP2A in MAPT cells. Mean ± SEM, *n* = 3; ^*∗*^*p* < 0.05, compared with the N2a group; #*p* < 0.05, compared with the MAPT group. CIG (M):100 *μ*g/mL, CIG (H): 200 *μ*g/Ml, MOR: morroniside (240 *μ*M), LOG: loganin (120 *μ*M), ML: morroniside (240 *μ*M) and loganin (120 *μ*M), CIG: cornel iridoid glycosides, and MAPT: microtubule-associated protein tau.

Morroniside and loganin account for 70% of the effective ingredients of CIG. To verify if morroniside and loganin monomers alone would have the same effect as CIG, we examined the calpain inhibition effect of these components in isolation and in combination. MAPT cells that were treated with morroniside and loganin showed results equivalent to those found for medium-dose CIG (100 *μ*g/mL). Morroniside, loganin, and CIG at different concentrations were found to reduce the ratio (150 kDa/250 kDa) of spectrin *α* II caused by MAPT overexpression, which led to a decrease in calpain activity (Figure 3(a)). In combination, morroniside coordinated with loganin to reduce the high expression level of calpain 1 induced by MAPT overexpression (Figure 3(a)). SDS-PAGE results revealed that the previously low expression level of p-GSK-3*β* was significantly increased by the administration of CIG, morroniside, and loganin treatment (Figure 3(d)). Moreover, it was found that the activity of PP2A had significantly increased following treatment with morroniside, loganin, and CIG (Figure 3(f)).

### 3.5. Effect of CIG on Calpain, GSK-3*β*, and PP2A Activity in Calpain-Activated N2a Cells

**Figure 4:**
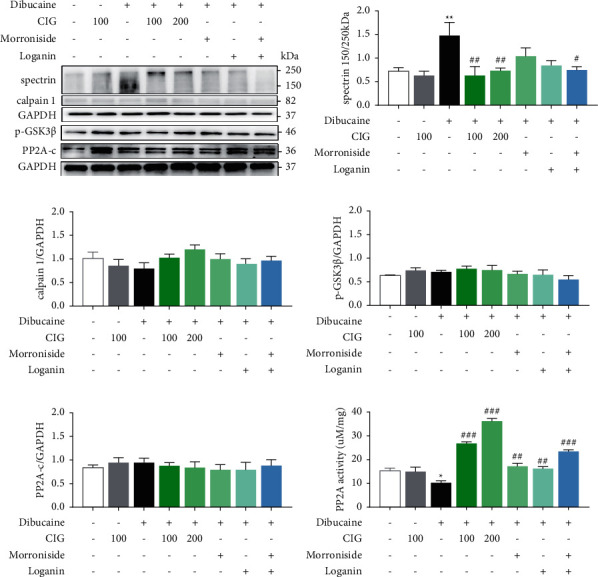
Effects of treatment with CIG, morroniside, and loganin on dibucaine-treated cells. (a) CIG reduced calpain activity in calpain-activated N2a cells. The expression of spectrin (b**) ***α* II, (c) calpain 1, (d) phosphorylation of GSK-3*β* at Ser 9, (e) PP2A-c, and GAPDH in dibucaine (200 *μ*M) led to the detection of calpain-activated N2a cells by western blots and semiquantitative analysis. (f) The activity of PP2A in dibucaine led to calpain-activated N2a cells. Mean ± SEM, *n* = 3; *∗p* < 0.05, *∗∗p* < 0.01, compared with the N2a group; #*p* < 0.05, ##*p* < 0.01, ###*p* < 0.001, compared with the dibucaine group. Morroniside (240 *μ*M), loganin (120 *μ*M), IG: cornel iridoid glycosides; GSK-3*β*: glycogen synthase kinase 3*β*; PP2A: protein phosphatase 2A; and GAPDH: glyceraldehyde 3-phosphate dehydrogenase.

Dibucaine is a known activator of calpain, and N2a cells treated with dibucaine were used to investigate whether CIG could reduce the activation of calpain in N2a cells under this condition. We observed that, after treating N2a cells with dibucaine for 6 h, spectrin *α* II was truncated, which was reverted after the administration of CIG and its main components, morroniside and loganin (Figure 4(a)). However, there was no change in the expression levels of calpain 1/2 and PP2A in each group (Figure 4(a)). SDS-PAGE results revealed that the dibucaine-induced low expression of p-GSK-3*β* was not significantly increased by the administration of CIG, morroniside, and loganin treatment (Figure 4(a)). In addition, compared with vehicle-treated N2a cells, PP2A activity was significantly decreased in dibucaine-treated N2a cells. The activity of PP2A was enhanced after the administration of CIG, morroniside, and loganin (Figure 4(f)).

## 4. Discussion

Studies have found an increase in the activation of calpain in the brain of patients with Alzheimer's disease (AD) [15–17]. In animal models of AD, inhibition of calpain 1 and 2 restores normal synaptic function and cognitive behavior and decreases amyloid plaques [18]. Inhibitors of calpain diminish the hyperphosphorylation of tau [19]. This experiment showed that CIG and its active ingredients morroniside and loganin could reduce tau hyperphosphorylation by regulating the activity of calpain.

The hyperphosphorylation of tau protein is recognized as one of the main pathogenic mechanisms of AD [20], being positively correlated with the degree of cognitive dysfunction. The microtubule-associated protein tau (MAPT)*∗*P301S mice model was used in this study because it exhibits brain activation of glycogen synthase kinase 3*β* (GSK-3*β*), protein phosphatase 2A (PP2A) activity inhibition, and protein tau hyperphosphorylation; the phosphorylation of tau is regulated by GSK-3*β* and PP2A. In this study, CIG reduced the activation of GSK-3*β* and enhanced the activation of PP2A in P301S mice and MAPT-overexpressing N2a cells. These results are consistent with those of previous observational studies, in which CIG could regulate tau phosphorylation by kinase activity [21].

GSK-3*β* is reported to be the primary tau kinase activated by calpain [6]. GSK-3*β* activity can be regulated by phosphorylation and through the truncation of GSK-3*β*. A previous study described N-terminal proteolysis as a novel way to regulate GSK-3*β*, and calpain inhibitors can prevent this truncation [6, 22]. In this study, we found that CIG could improve the phosphorylation of GSK-3*β* at site Ser 9. This is similar to the results of a previous study. We also found that the N-terminal cleavage of GSK-3*β* was improved after CIG administration. However, this result has not been previously described; previous studies have found that the truncation of GSK-3*β* at the N-terminus was not regulated by calpain [23]. A possible explanation for this divergence is that the N-terminal truncation of GSK-3*β* was not stable, and the product was rapidly degraded in in vitro experiments.

PP2A accounts for most of the dephosphorylation activities of tau protein [24]. Calpain is also shown to mediate the activity of the PP2A [4, 25]. In accordance with the present results, previous studies have demonstrated that CIG regulates the influence of calpain on PP2A activity.

The activity of calpain in the cerebrospinal fluid of patients with AD is significantly increased [26]. We found high calpain activity in P301S mice and the MAPT cell line. Consistent with the literature, this study found that CIG led to the activation of calpain activity, as revealed by the almost complete loss of 250 kDa full-length spectrin protein and the appearance of a major 150 kDa spectrin cleavage product that is specific to calpain activity [27, 28].

These Ca^2+^-regulated protease calpains, both 1 and 2, were abnormally activated and abnormally high in the brains of patients with AD. In this study, we found that calpain 1 in the model group (P301S/MAPT) was significantly higher than that in the normal group (nTg/N2a). These results support those of previous studies. However, calpain 2 did not show this tendency. This result may be explained by the fact that calpain 2 may have a role in a reaction in which calpain 1 does not participate [29].

Morroniside and loganin are the main components of CIG. To explore their effect on calpain, the cells were treated with the monomers in isolation and their combination. Morroniside attenuates okadaic acid- (OA-) induced tau hyperphosphorylation via PP2A activation [30]. Loganin has been found to exhibit significant neuroprotective effects and the ability to slow down the process of neurogenesis in AD [31]. We found that morroniside and loganin act on MAPT cells alone. They can inhibit the activity of GSK-3*β* and enhance the activity of PP2A, but there was no significant difference compared to the model group. When morroniside and loganin are used in combination, it can significantly reduce the activity of GSK-3*β* and enhance the activity of PP2A, with results equivalent to those of CIG. The activator of calpain, dibucaine, can activate calpain 1 and calpain 2 in cells [32–34]. We used this cell model and found that CIG, morroniside, and loganin could reduce the calpain activity induced by dibucaine. However, the expression of calpain 1 did not change. We also found that the combined use of morroniside and loganin has a better effect than that of the isolated components, with results equivalent to those of CIG. This suggests that morroniside and loganin may have an internal interaction; therefore, their combined use can enhance efficacy.

The results indicate that CIG can regulate its downstream signals GSK-3*β* and PP2A by regulating calpain activity to reduce the hyperphosphorylation of tau protein.

In conclusion, this paper describes the useful effects of CIG and its main components, morroniside and loganin, which could reduce tau phosphorylation through the calpain 1/GSK-3*β*/PP2A pathway. The present study provides new ideas for the development of drugs to revert tau hyperphosphorylation, one of the main pathological mechanisms of AD.

## Data Availability

Data used to support the findings of this study are available from the corresponding author upon reasonable request.
